# A Rare Case of Parotid Gland Tuberculosis

**DOI:** 10.1155/2021/7484812

**Published:** 2021-01-06

**Authors:** Deepshikha Singh, Sudhir Mishra

**Affiliations:** Department of Pediatrics, Tata Main Hospital, Jamshedpur 831001, India

## Abstract

Parotid gland tuberculosis is a very rare form of extrapulmonary tuberculosis, with less than 200 cases reported in literature. We describe a 10-year-old female who presented with a swelling in the left parotid region during the last month. CT scan neck revealed an abscess in the left parotid gland extending into the submandibular gland, muscles, and bone. Pus aspirated by FNAC showed acid fast bacilli in the ZN stain, and GeneXpert was positive for rifampicin-sensitive *Mycobacterium tuberculosis*. She was successfully treated with antituberculous therapy given for 6 months. Parotid gland tuberculosis, although rare, has a good prognosis with drug therapy. Surgery is rarely required.

## 1. Introduction

Parotid gland tuberculosis is a very rare form of extrapulmonary tuberculosis, with less than 200 cases reported in literature worldwide [1]. Here, we present a case with unilateral swelling in the parotid region and an underlying abscess. It was caused by tuberculosis and was successfully treated with antituberculous drugs.

## 2. Case Presentation

**Figure 1:**
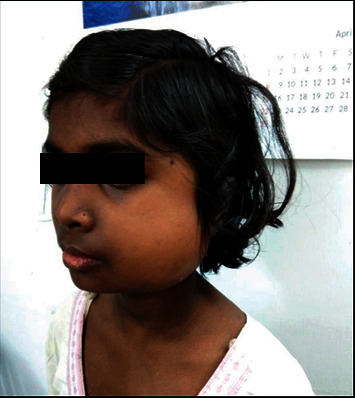
Left parotid region swelling in a 10-year-old girl.

**Figure 2:**
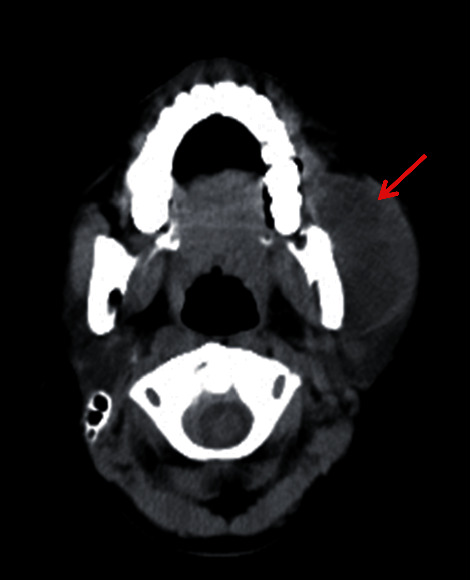
CT scan neck showing parotid abscess.

A 10-year-old female presented with gradually increasing swelling in the left parotid region during 1 month (Figure 1), associated with mild pain and difficulties in eating. There was no history of fever, cough, weight loss, trauma, or any other systemic symptoms and no history of contact with any tuberculosis patients. She had been living in a hostel for last 2 years. On physical examination, the patient had mild pallor, was thin built, and her weight was below 2 standard deviations for her age. On local examination of the swelling, a well-defined unilateral mass of 7 cm × 6 cm was found, obliterating the left angle of jaw. It was firm in consistency, mobile vertically, nontender with smooth skin surface, and had no discharging sinus. Multiple cervical lymph nodes were palpable about 1 cm in size, mobile, nonmatted. Right side angle of jaw was normal, with no lymphadenopathy anywhere else. Routine blood tests revealed hemoglobin 9.4 g/dl and total leucocyte count 5600/cumm with 40% neutrophils and 52% lymphocytes. ESR was 64 mm/hr. Ultrasonography of the swelling revealed a hypoechoic lesion with 180 cc volume, with septations and debris, suggestive of abscess. As there were no signs of inflammation clinically, a CT scan neck was done, which revealed abscess in the left temporalis and masseter muscle, left parotid, and submandibular gland, with destruction of anterior border of left ramus of mandible (Figure 2). FNAC was performed, and 3 ml pus was aspirated, which showed several scattered lymphonuclear cells, mixed with nuclear debris and some lipid vacuoles in a necrotic background. ZN stain showed scanty acid fast bacilli. Tuberculin test was highly positive at 22 mm. Chest X-ray was normal. GeneXpert on pus also came positive for *M. tuberculosis*. The patient was started on antituberculous therapy with 4 drugs, i.e., rifampicin, isoniazide, pyrazinamide, and ethambutol for 2 months, followed by rifampicin, isoniazide, and ethambutol for next 4 months [2]. On follow-up after 1 month, swelling size had reduced significantly and disappeared fully by 6 months. The patient gained 3 kg weight at the end of 6 months.

## 3. Discussion

The estimated incidence of tuberculosis in India was 2.69 million cases in 2018, accounting for one fourth of the global incidence [3]. Children up to 14 years of age accounted for 10% cases. Even in an endemic country, with such high incidence, tuberculosis of salivary gland is very rare, the most common presentation being pulmonary tuberculosis.

Parotid gland is a rare site of tubercular infection as the antibacterial activity of thiocyanate ions and proteolytic enzymes provide relative immunity, and there is less chance of stagnation due to salivary flow [4]. The parotid gland most commonly gets infected by direct extension of mycobacterium from the oral cavity via its duct [5]. Other less common routes are hematogenous spread and lymphatic spread from distant pulmonary focus [6]. Most common presentation is a unilateral painless parotid mass. Some cases are reported to have facial nerve palsy or a draining sinus at presentation [7].

First step in workup of a slow growing swelling of parotid gland is imaging, as benign tumours are the most common cause. Other differential diagnoses are parotid cyst, collagen vascular disorders like sarcoidosis, and rarely malignant tumours. Ultrasonography, CT scan, and MRI are the imaging modalities used, but all of them have nonspecific findings [8]. Two types of parotid involvement have been described in ultrasonography findings, the parenchymal type, where there is diffuse involvement of superficial part of the parotid gland, and periparotid type, where intra- and periglandular lymph nodes are affected and might present as an abscess later [9, 10]. In our case, there was abscess formation. CT scan also revealed involvement of the submandibular gland and bony destruction. There was no evidence of pulmonary tuberculosis. A similar case has been reported from India, reporting a 13-year-old girl who had a tubercular parotid abscess and a normal chest X-ray [9]. A metanalysis of 49 cases of parotid tuberculosis showed presence of pulmonary tuberculosis in only 25% cases [7]. Cases described early in literature were confirmed after histopathology of surgically excised gland [4], but many cases reported in the last decade have been diagnosed with parotid tuberculosis by fine needle aspiration cytology (FNAC) [4, 8, 11]. FNAC has good sensitivity (81%–100%) as well as specificity (94%–100%) [12]. Although classical histopathological picture of epithelioid granuloma with caseous necrosis was not found in our case, as there was abscess formation, diagnosis was confirmed by evidence of acid fast bacilli in Ziehl–Neelsen stain and detection of mycobacterial DNA in cartridge based nucleic acid amplification test (CBNAAT) also known as GeneXpert. CBNAAT is more sensitive than microscopy, provides result in 2 hours, and also detects resistance to rifampicin [13]. A study done in India reported CBNAAT positive in 77.7% of all suspected tuberculosis cases with lesions in the head and neck region, whereas microscopy with ZN stain detected only 16.6% cases. The sensitivity and specificity compared to gold standard test, i.e., culture, were 28.6% and 100%, respectively [14]. Various studies have reported a sensitivity ranging for 25% to 91.5% compared to culture in extrapulmonary tuberculosis cases [15].

Parotid gland tuberculosis has a good prognosis with drug therapy. Antituberculous drugs are given for 6 months, including 2 months of intensive phase with isoniazide, rifampicin, pyrazinamide, and ethambutol, followed by 4 months of continuation phase with isoniazide, rifampicin, and ethambutol. Cases reported in the last decade have all been treated successfully with drug therapy. Surgery is rarely required. Tuberculosis should be kept in mind while evaluating a solitary parotid mass, as it is a medically treatable condition, and surgery can be avoided.

## Data Availability

The patient's data used in this case report are included within the article.
